# From Social Distancing to Social Connections: Insights From the Delivery of a Clinician-Caregiver Co-mediated Telehealth-Based Intervention in Young Children With Autism Spectrum Disorder

**DOI:** 10.3389/fpsyt.2021.700247

**Published:** 2021-07-01

**Authors:** Sudha M. Srinivasan, Wan-Chun Su, Corina Cleffi, Anjana N. Bhat

**Affiliations:** ^1^Physical Therapy Program, Department of Kinesiology, University of Connecticut, Mansfield, CT, United States; ^2^Department of Physical Therapy, University of Delaware, Newark, DE, United States; ^3^Biomechanics and Movement Science Program, University of Delaware, Newark, DE, United States; ^4^Department of Psychological and Brain Science, University of Delaware, Newark, DE, United States

**Keywords:** telehealth, autism spectrum disorder, clinician-caregiver co-mediated intervention, creative movement and play, virtual intervention

## Introduction

The COVID-19 pandemic and efforts directed toward containing virus spread have led to significant disruptions to children's lives worldwide due to school closures, lockdowns, quarantines, reduced access to healthcare services, limited socialization, and significant reduction in opportunities to engage in physical activity (1, 2). The effects of the pandemic are more severe in children with intellectual and developmental disabilities including Autism Spectrum Disorder (ASD) (3–5). For families of children with ASD, the pandemic presents a host of challenges including, (1) reduced/modified virtual access to educational and healthcare services (Applied Behavioral Analysis (ABA), occupational therapy (OT), social skills training, speech language therapy (SLT), etc.) required to manage children's complex symptoms/comorbidities, (2) disruptions in structured routines, stay-at-home orders, and the unpredictability of the pandemic coupled with a lack of understanding of the world-wide crisis, leading to an aggravation of children's behavioral symptoms and increase in anxiety/distress, (3) difficulties complying with pandemic mitigation efforts of social distancing, limited outdoor activities, hand washing, mask wearing, etc. due to ASD-related cognitive, social, and sensory impairments, and (4) increased parental stress due to concerns for family's health, juggling multiple home-, work-, and caregiving/homeschooling-related responsibilities, as well as due to the economic, social, and psychological effects of the pandemic (6–9). Overall, the confluence of multiple pandemic-related stressors places significant strain on the family unit of children with ASD and deserves special consideration to address these impacts.

Over the past year, several countries have shifted to telehealth approaches to address the urgent educational and healthcare needs of citizens (10–13). Telehealth involves the use of electronic information and telecommunication technologies to support long-distance clinical health care delivery, patient health-related education, and public health (14). This includes synchronous, real-time video conferencing between families and clinicians as well as asynchronous transmission of content (instructional modules, videos/images, and websites) via the internet for caregiver coaching (15). The advantages of telehealth over traditional face-to-face (F2F) service delivery include cost-effectiveness, expanded geographic access, reduction in family and clinician travel costs, freedom to learn content at own pace (for asynchronous content), and reduced chances of infection due to in-person contact during the pandemic (16, 17). There is growing evidence for the use of telehealth-based service delivery in children for ABA-based services, OT, and SLT (18–22). A systematic review of 28 studies that provided telehealth-based ABA training to interventionists suggested that all studies found improvements in at least one outcome measure including challenging behaviors, social communication, and imitation skills in children with ASD as well as increases in procedural fidelity/skills of interventionists; however, included studies were methodologically weak necessitating more rigorous research before telehealth can be accepted as evidence-based practice in ASD (23). Another review of 14 studies in ASD suggested that telehealth-based services were comparable in efficacy to F2F interventions and significantly superior to control groups who received either no intervention or only self-directed training (24). Overall, telehealth seems to be a promising modality that can complement in-person practice, and in certain situations (i.e., the ongoing pandemic), even serve as an alternative to regular therapeutic practice (25).

Evidence on telehealth in ASD has mostly focused on training parents to facilitate their child's development (12, 23, 26). Involvement of parents in their child's therapy has clear advantages: (1) parents are their child's first teachers, (2) they are highly invested in their child's progress and can provide unique insights regarding their child's strengths and weaknesses, (3) their involvement makes the intervention ecologically valid, (4) parent coaching can train them to identify and harness teachable opportunities outside therapy sessions to promote target skills, and finally, (5) this approach facilitates generalization and maintenance of learned skills and also reduces parental stress (16, 27). However, self-directed parent training in addition to therapist coaching leads to greater improvements in children's skills, parent self-efficacy, as well as parental satisfaction and acceptance of telehealth programs compared to self-directed training alone, suggesting the importance of expert involvement (13, 16, 28). Next, we report preliminary insights from our ongoing randomized controlled trial (RCT) that assesses the effects of a telehealth-based, caregiver and clinician co-mediated, creative play intervention in school-age children with ASD. Our experiences also have implications for similar interventions in younger children with ASD. With the onset of the pandemic, our research team had to rapidly modify our in-person intervention protocol to be delivered virtually through videoconferencing platforms. The transition to telehealth-based research presented its set of challenges. Nevertheless, like any cloud has a silver lining, our experiences indicate that the transition to a clinician-caregiver collaborative virtual treatment approach makes our research more pragmatic and family-centric (29).

## Telehealth-Based Creative Play Intervention

Our multi-site RCT compares the effects of a whole-body creative movement and play-based intervention with a seated play intervention on social communication, executive functioning, imitation, motor coordination, and social synchrony skills of children with ASD between 5 and 15 years. The broader goal of this project is to assess the utility of whole body, socially-embedded movement interventions in addressing both primary impairments and secondary co-morbidities in children with ASD. Our past work has suggested that music and movement-based “rhythm” interventions lead to an increase in socially-directed verbalization, imitation, and interpersonal synchrony skills, and also afford high levels of social attention and positive affect/smiling during training sessions (30–33). In the current study, we aim to replicate and further expand on our previous work by assessing the effects of a creative movement intervention combining elements of music, dance, and yoga in a larger sample of children with ASD across 2 different study sites. Children are matched on age, gender, and functional level and then randomly assigned to receive either the experimental “play” intervention or the control “seated play” intervention. The creative play group engages in music- and imitation-based, movement and social games including, (1) singing and ice-breaker activities, (2) action songs that focus on hand movements, (3) music making with instruments, (4) locomotor games, and (5) solo/partner yoga poses and breathing. The seated play group engages in standard-of-care, OT and special education-based activities including, (1) greetings and farewells, (2) reading story books, (3) fine motor games, (4) building activities using Play-doh® and Legos^TM^, and (5) art-craft activities. The program has built-in opportunities for imitation, turn taking, call and response, and creative improvisation. Prior to the pandemic, training was conducted in a small group setting involving the child, clinician, and an adult confederate. As a facilitator, the clinician provided task instructions, activity demos, corrective feedback, prompting, and reinforcement, as well as used behavioral strategies to ensure task compliance. The adult confederate was the child's “buddy model” and supplemented the clinician's efforts by partnering with the child during games, providing motivational support, and hand-on-hand assistance as needed. With the transition to telehealth-based research, we have expanded our small group to also include the child's caregivers (parent &/or siblings). While the clinician and adult confederate serve as the child's “virtual buddies,” caregivers serve as their “in-person” buddies during online training sessions.

In our experience so far of working with 9 families of children with ASD through telehealth, we find that a collaborative caregiver and clinician co-mediated effort works really well. Our insights are also supported by other research that suggests that the telehealth-model of intervention delivery requires greater active participation from parents to ensure intervention success compared to F2F delivery (16). Parents in fact report greater beneficial effects of having ongoing, synchronous clinician input and assistance compared to a purely self-directed or asynchronous model of telehealth (16, 17, 20). Below we provide further explanation of why a clinician-caregiver co-mediated approach has salient benefits compared to other modes of intervention delivery.

From the clinician's perspective, a collaborative approach is beneficial as they can observe the child during real-world interactions and provide valuable feedback/suggestions to parents. For children with ASD requiring moderate-to-high levels of support or for younger children, telehealth automatically heavily engages the caregiver to build and facilitate the clinician-child relationship. Although the clinician and adult confederate continue their instructional and motivational roles, the caregiver primarily assumes the following responsibilities: serving as the child's in-person, social, behavioral, and motor role- model and providing reinforcement, prompting, and manual assistance as needed during activities. Based on caregiver insights of their child's strengths and difficulties, clinicians and caregivers can collectively develop meaningful intervention goals for the child, design activities that provide the optimal, “just-right” challenge, tailor activities to suit child preferences, and plan treatment progression based on ongoing child response monitoring. A clinician-caregiver co-treatment approach allows a truly family-centric training program (a continuation of the early intervention model) with ongoing feedback from caregivers and also empowers caregivers for an easier translation of training principles and strategies into the child's daily routines.

From the caregiver perspective, anecdotal data from our study collected through structured exit interviews with caregivers at the end of the study suggest that they appreciate the opportunity to engage in enjoyable, collaborative movement games with their child within a small social group context. Such activities naturally afford abundant opportunities to promote joint attention, sharing of supplies, spontaneous and responsive social bids, turn taking, imitation, and social-motor attunement/synchrony in their child. The mutual clinician-caregiver relationship allows collective planning of training goals, activities, and strategies and also allows caregivers to learn effective strategies from clinicians and observe their child's progress over time. Moreover, involvement of siblings during training promotes family bonding and generalization of learned skills to broader family interactions. With pandemic-induced heightened stress levels, caregivers frequently confide in clinicians during and even beyond the training period regarding challenges faced in caring for their child. They report this to be a beneficial social connection as they cope with their ongoing struggles during these stressful times.

From a child's perspective, interacting remotely may be less intimidating than in-person interactions for some children (8). For others, particularly younger children, virtual interactions might in fact be difficult to engage in, especially given the requirements of focusing on the small laptop screen, the physical and social disconnect compounded by internet connectivity issues, and the need to follow remotely-delivered instructions. However, over the past year, many children have become used to online schooling and virtual interactions with remote instructors. In our experience, for a subgroup of children with more severe impairments and inattention, telehealth may not be an ideal model to promote target skills and may increase parental stress levels. For younger children, we find that use of principles from naturalistic developmental behavioral interventions (34) including tying training activities to children's interests (e.g., cartoon characters, favorite movies, etc.), incorporating multiple, competitive goal-oriented games, and caregiver modeling helps to engage children during training. Overall, virtual therapies are ideally suited for children requiring low-to-moderate support who are able to attend at least briefly to virtual partners during training sessions.

## Discussion: Challenges, Recommendations, and Future Directions

Although our initial results pertaining to implementation feasibility, fidelity, and familial acceptance of a telehealth-based intervention are promising, there are several challenges with this approach (see Table 1). For instance, the virtual mode has specific hardware, software, and connectivity requirements that some families and healthcare providers find challenging. There are also some inherent limitations with videoconferencing software: audio interruptions when multiple people speak/sing simultaneously, difficulties with transmission of audio clips/sounds across platforms, and drowning out of non-human audio sounds (e.g., musical instrument sounds). The training also requires significant parental buy-in in terms of patience and additional setup time for problem-solving the technology and the software settings as well as time and effort dedicated toward being part of the intervention. Moreover, for younger children, the success of the intervention hinges on parental involvement to provide demonstrations and prompting/assistance during training and further use learned interaction strategies/training activities with their child during and outside the training context. For children of any age requiring more support, the tele-therapy model may in fact lead to growing child frustration and parental stress (12, 13).

**Table 1:** Challenges and potential solutions during telehealth sessions.

**Challenges**	**Potential Solutions**
**Technical issues**	
Lack of equipment	- A kit is mailed to caregivers which includes a microphone and a webcam that is already mounted on a tripod ready-to-use by families. - If a family does not have access to a laptop, we provide a laptop with pre-loaded videoconferencing software.
Setup and troubleshooting of hardware and software	- A step-by-step installation guide for hardware equipment (webcam, tripod, and microphone) and video-conferencing software setup including written instructions, snapshots, and instructional videos are provided via email. - Caregivers are guided through the actual hardware and software setup process during the tech-support session conducted via a phone call. - Following successful setup of the hardware and software, the family is sent an email with a password-protected Zoom link for a live session. - All audio and video settings are tested and optimally configured during this live session in preparation of the next virtual session with the child.
Connectivity issues	- If the family is facing streaming issues during the test video-call using video-conferencing software, we try to identify the area of their house where they may have the best internet access and test the quality of the connection from that location. - If needed, caregivers may be asked to limit the number of devices using the internet at the time of the scheduled call with the research team to ensure better streaming speeds and audio/video quality during the call.
Type of view on videoconferencing software	- Caregivers are provided information on different view types (e.g., gallery and speaker views) during the test video-call and the research team recommends the ideal view to be used during the intervention sessions. - Specifically, caregivers are asked to try out different viewing options during the virtual tech-support session so that they are familiar and comfortable changing these settings. - Caregivers are recommended to use the gallery/grid view during group training sessions (so that child can see all participants in the session) and speaker/focus view during fine motor activities using small objects.
**Participant issues**	
Setup of environment	- The caregiver in collaboration with the research team identify a quiet, distraction-free area during the tech-support session that can be reserved for training sessions. - Caregivers are requested to adjust furniture and remove any items that block views to ensure that both the child and caregivers are visible throughout the training sessions. - As mentioned above, caregivers are guided to figure out optimal camera position and room lighting in the reserved space for testing/training.
Child/or caregiver not in view	- Adjustments to camera position are made by the caregiver in an ongoing manner during the session to ensure that the child is always in full view of the camera.
Child/caregiver not heard	- Caregivers are reminded to turn on the microphone at the start of every session and we request them to place it as close to the child as possible to ensure optimal sound quality. - If it is hard to hear the child's responses, we always ask the caregivers for clarification on what the child is trying to communicate. Caregivers are also encouraged to intimate the clinician/confederate if they observe any non-verbal communicative behaviors (pointing, signs for “more,” “all done,” etc.) that the clinician/confederate may have missed.
Clinician not seen/heard	- To ensure that the clinician/confederate are appropriately visible and heard, they also use a tripod mounted webcam and a microphone at their end.
**Intervention-related issues**	
Clinician-played music not heard	- To allow music played on the clinician's laptop to be transmitted through video-conferencing software and be audible to the child/caregiver, we enable settings in the video-conferencing software that allow the clinician to “share sound.” - The clinician uses a speaker to ensure adequate amplification of played sound so that it can be heard loud enough at the child's end. - In addition, the music files are also sent to caregivers ahead of time of the session. In case caregivers are having trouble hearing the music, they are asked to play these music files at their end during the sessions.
Unclear expectations regarding sessions	- We use picture boards to clearly indicate the activities for the day and transitions between activities to the children. - We make a behavioral agreement with the child at the start of each session using a rules sheet that uses words and pictures to list do's and don'ts for the session. The child is provided an intermittent reminder of the rules as needed during the rest of the training session to ensure the child's compliance.
Clinician movements/training activities not clear	- The clinician and the adult confederate ensure that their movements are large and exaggerated in amplitude to be clearly visible to the child and their caregiver. - The clinician, confederate, and the child/caregiver have identical kits of training supplies. This allows children to better follow the instructional bids of the clinician/confederate using supplies/props. - Instructions are provided in a multimodal format, i.e., we show children pictures of movements to be practiced, the virtual and in-person partners provide a visual demonstration of movements, we use short verbal descriptors of movements such as “tap and clap,” “hands up and down” to cue key movement components during our demonstration and during the child's practice, and the caregiver may also provide manual assistance or physical prompting as required by the child during movements/activities.
Child running away during sessions	- We set clear expectations with the child about session structure at the outset of the session by using a visual schedule and by going through a rules sheet for the session. - During sessions, the child is encouraged by the clinician to clearly communicate gesturally/verbally/using pictures if they need a break. - We solicit parental input on strategies to engage the child, for e.g., call-response ideas such as “macaroni & cheese… everybody freeze” (to get the child to stay on their spot), or use of phrases such as “eyes on John” (to get the child to focus on their virtual partner), or showing pictures of cartoon characters doing exercises (to motivate the child to exercise with their favorite character), etc. - The session is structured to include short 3–5 min activity bursts followed by opportunities for scheduled 30 sec to 1 min breaks if required by the child. - We work with the family to identify the child's familiar/preferred reinforcement system and adopt it during our sessions, for example, token economy, stickers, quick iPAD break, etc.
Child inattention toward laptop screen	- Caregiver is asked to provide a visual model of ideal interactions with virtual partners. - The clinician and confederate use loud voices and clear, brief instructions to solicit and sustain child's attention. - During whole-body activities, there are built-in times between activities when caregivers and the child are asked to sit down in front of screen to observe the movement demo provided by the clinician/confederate, or to engage in conversations/social exchanges, or to see pictures of activities that will be practiced next, or power point slides of their favorite cartoon characters encouraging them to practice training activities. - The clinician/confederate regularly (typically after every activity) initiate gestural reinforcement bids such as virtual high-fives, fist bumps, etc. where children are typically asked to come to the screen and give high-fives to their virtual partners. - We also use call-response strategies to solicit child's attention: e.g., “Hocus-focus… time to focus,” etc. - We use playful games with clear functional goals (improve accuracy, timing, speed, etc. of movement performance) to challenge the child.
Inadequate practice of training activities	- Caregivers are provided a list of online resources (short YouTube videos) and parent training activities every week that are tailored to their child's interests to facilitate practice of similar activities beyond the training sessions. - Caregivers are also sent email or text reminders to practice activities with their child each week. These activities are also documented in a weekly training diary that is filled out by researchers in collaboration with caregivers at the end of the training sessions.

Presently, a glimmer of light exists at the end of the COVID tunnel. Widespread vaccinations over the next several months may help us slowly transition back to a socially-close world resembling the pre-pandemic state. As we return to conventional F2F research, our learnings over the past year clearly suggest that telehealth-based research can serve as a valuable complement to F2F research even beyond the pandemic. Telehealth-based settings afford greater involvement of the child's family during training, provide opportunities to use household training supplies, and allow practice of activities within naturalistic settings, making the training truly ecologically valid. These factors, coupled with the economic and geographic access-related advantages of virtual interventions, may lead to some families preferring to engage in virtual research exclusively (25, 35). Therefore, it would be prudent for researchers to provide families with the flexibility to choose between F2F interventions with adequate precautions and virtual training sessions (15, 17, 36). Although this adds variability to collected data, meticulous documentation of intervention format-related variables (F2F vs. virtual, clinicians alone or clinicians + caregivers) can allow researchers to systematically assess the effects of these variables on treatment effects (35). Moreover, researchers should consider tailoring the level of parental involvement based on the child's abilities; for instance, verbal children with ASD may be able to independently participate in virtual sessions, whereas low-verbal and younger children may require greater parental support (12). Although there is preliminary evidence in support of the equivalence of F2F and telehealth-based training approaches in ASD (24, 37), more rigorous research is needed on this topic. Overall, our experiences suggest that a collaborative clinician-caregiver co-mediated, telehealth-based intervention is a feasible, ecologically valid, and acceptable modality to promote social communication, behavioral, and motor skills in children with ASD.

## Author Contributions

SS was involved in the conception and writing of the first draft of the manuscript. All authors were involved in proof-reading and editing of the manuscript.

## Conflict of Interest

The authors declare that the research was conducted in the absence of any commercial or financial relationships that could be construed as a potential conflict of interest.
